# Prevalence and risk factors for *Clostridium difficile* infection: a retrospective analysis

**DOI:** 10.1186/1471-2334-13-S1-P45

**Published:** 2013-12-16

**Authors:** Mirela Indries

**Affiliations:** 1 Municipal Clinical Hospital "Dr. Gabriel Curteanu" Oradea, Romania

## Background

*Clostridium difficile* infection (CDI) is the most common cause of nosocomial infection and it is associated with increasing morbidity and mortality. The study aimed to evaluate the CDI incidence and the relapse associated risk factors in our service.

## Method

This is a retrospective analysis of the cases with *Clostridium difficile* infection from 01 January 2012 to 31 July 2013, admitted to the Infection Diseases Department. Demographic and clinical data and risk factors (antibiotic use, underlying malignancy, chemotherapy, corticosteroids, proton-pump inhibitors – PPI – use) were noted.

## Results

In the above-mentioned time span, there were 46 cases of patients with CDI. We excluded all cases where data were not sufficient to support CDI diagnosis. Out of the total cases, 58.69% were female, 54.34% from Oradea. Most cases were elderly people, 31 of them (67.39%) over the age of 56, and 63.04% had a recent history of surgery or in-hospital treatment. Over 32% followed antibiotic treatment and/or PPI at home. Confirmation of the diagnosis was made by the quantitative (43 cases) or qualitative toxin A & B (4 cases). There were different forms of the disease, ranging from mild gastroenteritis to colitis, pseudomembranous colitis and toxic megacolon, some with fatal outcome (1 case).Therapy included rifaximin and metronidazole (oral) for mild cases (11 cases, 23.91%), glycopeptides (oral) ± metronidazole oral/intravenously for moderate or severe cases. There were 11 cases with recurrence (23.40%).

## Conclusion

The CDI incidence increased dramatically in our service in the first semester of 2013 compared to 2012. The prolonged hospitalization is a risk factor for the occurrence of CDI recurrence.

